# Clinical outcomes of subcutaneous vs. transvenous implantable defibrillator therapy in a polymorbid patient cohort

**DOI:** 10.3389/fcvm.2022.1008311

**Published:** 2022-10-18

**Authors:** Badder Kattih, Felix Operhalski, Felicitas Boeckling, Florian Hecker, Felix Michael, Mate Vamos, Stefan H. Hohnloser, Julia W. Erath

**Affiliations:** ^1^Division of Clinical Electrophysiology, Department of Cardiology, University Hospital Frankfurt, J. W. Goethe University, Frankfurt am Main, Germany; ^2^Department of Cardiac Surgery, University Hospital Frankfurt, J. W. Goethe University, Frankfurt am Main, Germany; ^3^Cardiac Electrophysiology Division, Department of Internal Medicine, University of Szeged, Szeged, Hungary

**Keywords:** S-ICD, TV-ICD, implantable cardioverter-defibrillator (ICD), sudden cardiac death, subcutaneous ICD, transvenous ICD

## Abstract

**Background:**

The subcutaneous implantable cardioverter-defibrillator (S-ICD) has been designed to overcome lead-related complications and device endocarditis. Lacking the ability for pacing or resynchronization therapy its usage is limited to selected patients at risk for sudden cardiac death (SCD).

**Objective:**

The aim of this single-center study was to assess clinical outcomes of S-ICD and single-chamber transvenous (TV)-ICD in an all-comers population.

**Methods:**

The study cohort comprised a total of 119 ICD patients who underwent either S-ICD (*n* = 35) or TV-ICD (*n* = 84) implantation at the University Hospital Frankfurt from 2009 to 2017. By applying an inverse probability-weighting (IPW) analysis based on the propensity score including the Charlson Comorbidity Index (CCI) to adjust for potential extracardiac comorbidities, we aimed for head-to-head comparison on the study composite endpoint: overall survival, hospitalization, and device-associated events (including appropriate and inappropriate shocks or system-related complications).

**Results:**

The median age of the study population was 66.0 years, 22.7% of the patients were female. The underlying heart disease was ischemic cardiomyopathy (61.4%) with a median LVEF of 30%. Only 52.9% had received an ICD for primary prevention, most of the patients (67.3%) had advanced heart failure (NYHA class II–III) and 16.8% were in atrial fibrillation. CCI was 5 points in TV-ICD patients vs. 4 points for patients with S-ICD (*p* = 0.209) indicating increased morbidity. The composite endpoint occurred in 38 patients (31.9 %), revealing no significant difference between patients implanted with an S-ICD or TV-ICD (unweighted HR 1.50, 95 % confidence interval (CI) 0.78–2.90; *p* = 0.229, weighted HR 0.94, 95% CI, 0.61–1.50, *p* = 0.777). Furthermore, we observed no difference in any single clinical endpoint or device-associated outcome, neither in the unweighted cohort nor following inverse probability-weighting.

**Conclusion:**

Clinical outcomes of the S-ICD and TV-ICD revealed no differences in the composite endpoint including survival, freedom of hospitalization and device-associated events, even after careful adjustment for potential confounders. Moreover, the CCI was evaluated in a S-ICD cohort demonstrating higher survival rates than predicted by the CCI in young, polymorbid (S-)ICD patients.

## Introduction

Sudden cardiac death (SCD) is reported to account for 30% of all cardiovascular death causes in Germany taking 65.000 lives per year (1). Implantable cardioverter-defibrillators (ICD) have been proven to efficiently prevent sudden cardiac arrhythmic death in pivotal trials (2–4).

Advances in ICD programming have reduced the burden of shocks, but lead-related complications remain an unalterable drawback of transvenous implantable cardioverter-defibrillator (TV-ICD) therapy, resulting in significant morbidity (5). Transvenous sensing and defibrillation leads are associated with both infective and mechanical complications, such as endocarditis, pneumothorax, venous occlusion, lead fracture, and cardiac perforation (6).

The subcutaneous implantable cardioverter-defibrillator (S-ICD) has been designed to overcome lead-related complications and device endocarditis lacking the ability for pacing or resynchronization therapy and can therefore be used only in selected patients (7). Current American and European guidelines recommend S-ICD therapy as a class IIa indication in patients without indication for pacing, cardiac resynchronization or anti-tachycardic pacing (8, 9).

Observational studies demonstrated clinical efficacy of the S-ICD with an initial high inappropriate shock rate up to 13 % due to limited discrimination abilities (10, 11). Although the rate of inappropriate shocks seems to be lower in patients implanted with S-ICD and channelopathy in a substudy of the EFFORTLESS trial (12). The Prospective Randomized Comparison of Subcutaneous and Transvenous Implantable Cardioverter Defibrillator Therapy (PRAETORIAN) trial evaluated 849 patients with primary preventive ICD indication lacking the indication for pacing who were randomly assigned to receive either a TV-ICD or S-ICD demonstrating non-inferiority of the S-ICD regarding inappropriate shocks and device-related complications (13). Although this is the first randomized controlled trial to evaluate S-ICD and TV-ICD patients, a significant proportion of S-ICD and TV-ICD candidates have been excluded a priori (e.g., patients with history of device-associated complications or secondary prevention indication for SCD). The UNTOUCHED trial (Understanding Outcomes With the S-ICD in Primary Prevention Patients With Low Ejection Fraction) was designed to evaluate the inappropriate shock rate in a more typical, contemporary S-ICD cohort (14). In this study, 1,111 patients were implanted with an S-ICD. Due to optimized programming algorithms and application of filters (e.g., the smart pass filter to overcome T-wave oversensing), the inappropriate shock rate was 3.1% year (14). Of note, only patients with primary prevention indication for SCD have been included (14). Thus, there is a need for real-world data to investigate whether results of these studies can be extrapolated to daily clinical practice (15, 16). In the present study, we aimed to investigate clinical outcomes in an all-comers cohort of patients with primary and secondary preventive indication for ICD therapy, and also patients who were implanted with a previous defibrillator, receiving either a single chamber TV-ICD or a S-ICD.

## Methods

### Patient population

This retrospective observational cohort study is based on data of 192 consecutive patients either implanted with a single chamber TV-ICD (*n* = 140) or a S-ICD (*n* = 52) at the Frankfurt University Hospital, Division of Cardiology from 2009 to 2017. Seventy-three patients were excluded from analysis due to missing data to apply propensity score adjustment resulting in 119 patients included in the final study cohort. The devices used were S-ICDs (Boston Scientific, Marlborough, Massachusetts) and TV-ICDs (Biotronik, Berlin, Germany; Boston Scientific; Medtronic, Dublin, Ireland; and St. Jude Medical, Saint Paul, Minnesota). The majority of TV-ICD patients were implanted under local anesthesia, while most of the S-ICD patients received analgosedation in preparation for DFT testing. Patient demographic data were abstracted from the patient files. All patients consented to data use for quality and research purposes. The study was approved by the IRB of the J.W. Goethe University and conforms to the ethical guidelines of the Declaration of Helsinki.

### Data collection and follow-up

Data were retrospectively collected from the index hospitalization at the time of the initial S-ICD / TV-ICD implantation and at each follow-up visit which took place every 6 months or at the time of unscheduled visits in the out- or in-patient clinic. Data collection included patient characteristics such as age, indication for defibrillator therapy, echocardiographic data [e.g., left ventricular ejection fraction (LVEF)], and relevant cardiovascular and non-cardiac comorbid conditions. ECG parameters such as atrioventricular (AV) conduction and QTc were additionally assessed. NYHA-classification was assessed at implantation and every follow-up visit. Pertinent medication use (heart failure medication, statins, and antiarrhythmic drugs) was documented. To correct for potential extracardiac comorbidities, the Charlson Comorbidity Index (17) was calculated for every patient. This index incorporates 19 primary diseases and the patient's age by a point system. The higher the calculated score, the lower the one-year survival rate (17). All relevant information was entered into a customized database. For missing data, particularly in case of missed follow-up visits, family members, treating physicians, or other hospitals were contacted to retrieve the missing information.

### Study endpoints

Our study data were primarily evaluated on a composite endpoint (overall survival, freedom of hospitalization, freedom of device-related events) following inverse probability of treatment weighting. For explorative purposes overall survival, freedom of hospitalization and freedom of device-related events were also assessed individually without adjustment for multiple comparisons to characterize the study population in the unadjusted and adjusted study cohort and to report its comparability with previous studies. Freedom of device-related events was calculated from time to inappropriate therapy, time to appropriate therapy and time to first system infection. Appropriate therapy was defined as shocks for ventricular tachycardia (VT) or ventricular fibrillation (VF). Inappropriate therapy consisted of shocks for heart rhythms other than VT or VF. Kaplan-Meier method followed by Cox proportional-hazards regression were performed to report the outcomes in the unweighted or with inverse probability weighting (IPW) in the weighted study cohort. Further, rate of appropriate and inappropriate device discharge and device-associated complications was provided.

### Statistical analysis

**Figure 1:**
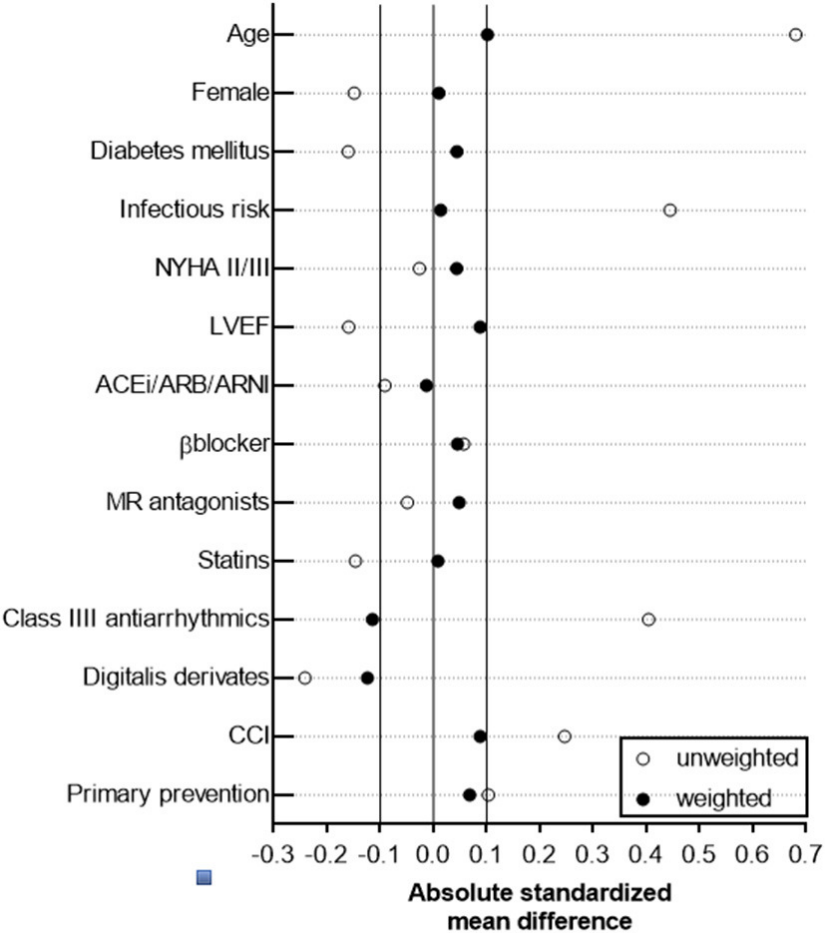
Forrest plot for standardized mean differences of covariates included in the IPW model.

Based on the non-randomized nature of this retrospective observational cohort study, an established statistical technique (propensity score method) was applied to yield a balanced distribution of baseline characteristics in the study cohort and to allow direct head-to-head comparison of the study outcome parameters between TV-ICD and S-ICD, which has been widely used in perioperative and cardiovascular clinical trials (18–22). We preferred a propensity score-based method, which retains the patient data and creates a pseudo population with an optimal covariate balance, over other statistical methods (e.g., conventional multivariable regression methods) to improve adjustment for measured confounders in a small dataset and to address potential confounding by indication (TV-ICD vs. S-ICD) when using observational data. Indeed, inverse probability weighting based on the propensity score is an established approach to deal with potential confounding factors in observational studies and for confounding by indication (23, 24). By applying the inverse probability weighting method, individual patients of the original study population (*n* = 119) were differentially weighted, thus resulting in a statistical pseudo population with simulated additional observations (*n* = 231) in which baseline patient characteristics in the weighted S-ICD (*n* = 111) and TV-ICD (*n* = 120) group are balanced (24–27). Briefly, the propensity score was calculated using a logistic regression model, in which the type of ICD (TV-ICD or S-ICD) has been regressed as dependent variable on relevant baseline characteristics (28). Corresponding weights for patients in the S-ICD group were calculated by $\frac{1}{PS}$ and for those in the TV-ICD by $\frac{1}{1-PS}$ as previously described (29). Weights were incorporated in subsequent analyses comparing the cardiovascular study outcome parameter between both ICD groups, in which the distribution of measured confounding factors is independent of ICD type. Both, balance of measured and unmeasured covariates, is achieved only in randomized, placebo-controlled trials, which has to be taken into account when interpreting our results. Absolute standardized difference ≤ 0.1 for measured covariates suggested appropriate balance between the groups, except for usage of class III AAD and digitalis glycosides (Figure 1).

Statistical analysis was performed with SPSS statistical software version 27.0 (IBM). Analysis of data distribution was performed with the Kolmogorov–Smirnov and Shapiro–Wilk Test. Continuous variables are presented as median with interquartile range (IQR) or means with standard deviations (SD) based on data distribution unless otherwise noted. Categorical variables are provided with absolute numbers (n) and percentages (%). We used the students *T*-test or Mann Whitney U test (when appropriate) to compare continuous variables and the Pearson chi-square test or Fisher exact test to compare categorical variables in the unweighted cohort. Two-sided tests were used and *p* < 0.05 were considered statistically significant. To estimate confounder-adjusted KM survival curves with weighted log-rank testing, the R package *RISCA* (v0.8.2) was used (30, 31). Survival analysis and visualization was further facilitated using the R package *survminer* (v0.4.9) and *survival* (v3.1-8).

For exploratory purposes median follow-up time for survival was calculated according to the inverse Kaplan–Meier method.

## Results

### Patient characteristics

We analyzed a total of 119 patients either implanted with a single-chamber TV-ICD or S-ICD. The S-ICD group comprised 35 (29.4 %) patients, while 84 patients (70.6 %) were included in the TV-ICD control group. During a median follow-up of 512 days (95 % CI, 228.5–795.5 days), the estimated 1.5-year overall survival rate in the study cohort was 95.0%. The baseline characteristics of both groups are summarized in Table 1. The median age of the entire population was 66.0 years, 22.7 % of the patients were female. Approximately two-thirds of the study cohort suffered from ischemic cardiomyopathy with a median left ventricular ejection fraction of 30%. Sixty-three (52.9 %) of the ICD systems were implanted for primary prevention. Most of the patients had New York Heart Association (NYHA) class II / III heart failure (67.3%), and 16.8% of the patients had atrial fibrillation. Patient characteristics were similar between both groups except for a higher age (*p* = 0.001) and differences in heart failure medication (*p* = 0.026) in the TV-ICD group, while infectious risk factors (*p* = 0.004) such as diabetes or oral immunosuppressive therapy were more prevalent in the S-ICD group. However, the standardized mean differences indicated further residual unequally distributed confounding factors (Table 1).

**Table 1:** Baseline characteristics of the unweighted and weighted study cohort.

	**Unweighted**				**Weighted**			
	**TV-ICD (*n* = 84)**	**S-ICD (*n* = 35)**	**SDM**	***p*-Value**	**TV-ICD (*n* = 120)**	**S-ICD (*n* = 111)**	**SDM**	***p*-Value**
Age, years	68 (55–77)	54 (43–71)	0.68	0.001	66 (48–76)	66 (50–74)	0.10	0.554
CCI, pts	5 (3–7)	4 (2–6)	0.25	0.209	5 (2–7)	4 (3–6)	0.09	0.528
Female, *n* (%)	20 (23.8)	7 (20.0)	−0.15	0.811	28 (23.6)	26 (23.9)	0.01	1.000
LVEF, %	30 (25–45)	30 (23–40)	−0.16	0.513	30 (25–45)	30 (25–35)	0.09	0.808
Diabetes mellitus, *n* (%)	26 (31)	9 (25.7)	−0.16	0.662	35 (29.1)	34 (30.7)	0.04	0.886
Infectious risks, *n* (%)	28 (33.3)	22 (62.9)	0.44	0.004	50 (41,6)	47 (42.2)	0.01	1.000
Prevention, *n* (%)			0.10	0.553			0.07	0.507
Primary	46 (54.8)	17 (48.6)			64 (53.8)	65 (58.5)		
Secondary	38 (45.2)	18 (51.4)			55 (46.2)	46 (41.5)		
NYHA class, *n* (%)			−0.03	0.800			0.04	0.262
I	24 (28.6)	10 (28.6)			32 (26.5)	27 (24)		
II	38 (45.2)	13 (37.1)			55 (45.8)	58 (52.9)		
III	19 (22.6)	10 (28.6)			28 (23.7)	17 (15.4)		
IV	3 (3.6)	2 (5.7)			5 (3.9)	9 (7.9)		
B-blockers, *n* (%)	76 (90.5)	34 (97.1)	0.06	0.279	111 (92.8)	109 (98.1)	0.05	0.061
Digitalis glycosides, *n* (%)	19 (22.6)	6 (17.1)	−0.24	0.625	25 (20.8)	20 (18)	−0.12	0.621
Class III AAD, *n* (%)	7 (8.3)	5 (14.3)	0.40	0.332	14 (11.4)	11 (10)	−0.11	0.833
ARB/ACEi/ ARNI, *n* (%)	83 (98.8)	31 (88.6)	−0.09	0.026	116 (97)	106 (95.5)	−0.01	0.741
MR antagonists, *n* (%)	56 (66.7)	22 (62.9)	−0.05	0.679	82 (68.5)	81 (72.7)	0.05	0.472
Statins, *n* (%)	60 (71.4)	21 (60.0)	−0.15	0.281	80 (67.2)	75 (67.9)	0.01	1.000
AV time, ms	169 (150–186)	160 (146–184)		0.312	168 (150–186)	160 (148–172)		0.049
QTc interval, ms	435 (416–461)	445.3 (410–460)		0.543	440 (417–464)	429 (410–460)		0.747
CCI, predicted 1-year survival, %	21 (0–77)	53 (2–90)		0.058	21 (0–90)	53 (2–77)		0.316
Heart disease, *n* (%)				0.494				0.482
Ischemic	55 (65.5)	18 (51.4)			76 (63.3)	67 (60.6)		
Dilated	17 (20.2)	10 (28.6)			26 (22.1)	30 (27.2)		
Congenital	3 (3.6)	1 (2.9)			6 (4.8)	2 (2)		
Other	9 (10.7)	6 (17.1)			12 (9.8)	11 (10.3)		
ECG rhythm, *n* (%)				0.404				0.086
Sinus rhythm	66 (83.5)	24 (72.7)			97 (85.5)	84 (77)		
AF	12 (15.2)	8 (24.2)			15 (13.4)	19 (17.6)		
Paced	1 (1.3)	1 (3.0)			1 (1)	6 (5.4)		
Obesity, *n* (%)	16 (20.8)	8 (22.9)		0.808	21 (19.8)	25 (22.2)		0.622
CKD, *n* (%)	19 (22.6)	8 (22.9)		1.000	28 (23)	24 (22)		0.540

TV-ICD, transvenous implantable cardioverter-defibrillator; S-ICD, subcutaneous implantable cardioverter-defibrillator; SDM, standardized mean difference; CCI, Charlson Comorbidity Index; LVEF, left ventricular ejection fraction; NYHA, New York Heart Association; AAD, antiarrhythmic drugs; ARB, angiotensin receptor blockers; ACEi, angiotensin-converting-enzyme inhibitors; ARNI, Angiotensin Receptor-Neprilysin Inhibitor; MR antagonists, mineralocorticoid receptor antagonists; AV time, atrioventricular time; ECG, electrocardiogram; AF, atrial fibrillation; CKD, chronic kidney disease; PAD, peripheral artery disease.

In the adjusted study population following inverse probability weighting based on the propensity score, an improved overall balance of baseline characteristics and standardized mean differences was achieved, indicating that the weighted study cohorts were comparable in important baseline characteristics (Figure 1 and Table 1).

### Clinical outcomes

#### Composite endpoint

**Figure 2:**
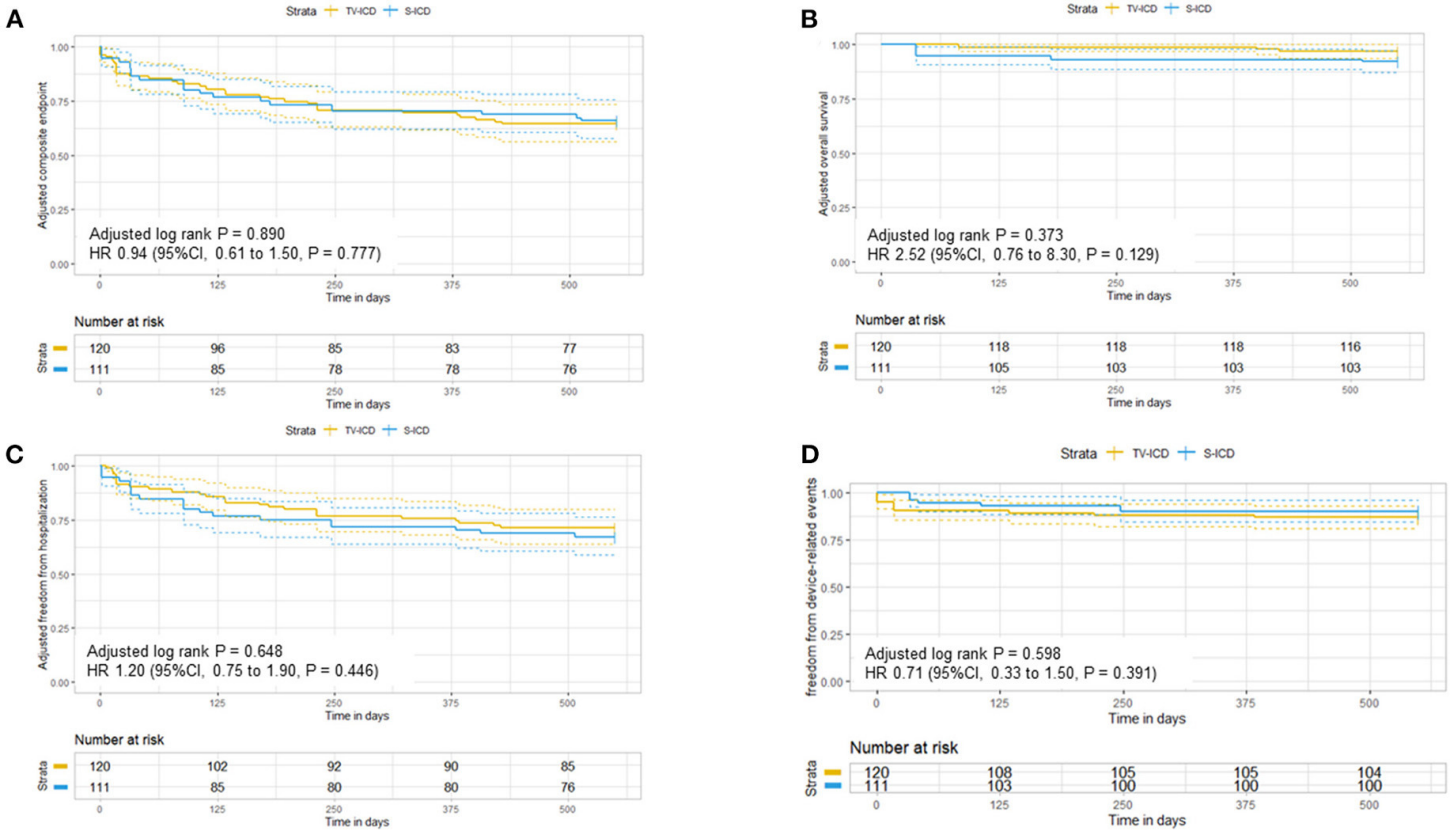
Adjusted Kaplan-Meier curves in patients with subcutaneous and transvenous ICD following inverse probability weighting for **(A)** the composite endpoint, **(B)** overall survival, **(C)** freedom of hospitalization, and **(D)** freedom of the device-related events at 1.5 year.

In the unweighted study group, no significant differences in the composite endpoint (survival, freedom of hospitalization, and freedom of device-related events) were observed between patients implanted with an S-ICD or TV-ICD (event number 14 vs. 24) over a follow-up time of 1.5 years using the Kaplan-Meier estimate (p = 0.226) or Cox-regression (HR 1.50, 95 % confidence interval (CI) 0.78–2.90; *p* = 0.229) (Supplementary Figure 1A). Importantly, the hazard ratio for the adjusted composite end point was 0.94 (95 % CI, 0.61–1.50; *p* = 0.777) without differences in the TV-ICD group and S-ICD group (p for log rank = 0.890) in the weighted study group (Figure 2A).

#### Survival

During the study follow-up time, a total of 6 patients (5.0%) died (3 TV-ICD / 3 S-ICD) in the unadjusted cohort. The main causes of death were cardiac non-arrhythmic. One S-ICD patient died due to electrical storm while living alone. Again, S-ICD therapy was not different to TV-ICD therapy in our study cohort concerning overall survival (HR 2.50, 95 % CI 0.5–12.0, *p* = 0.278) (Supplementary Figure 1B). These results were robust on the weighted analysis (HR 2.52, 95 % CI 0.76–8.30, *p* = 0.129) (Figure 2B).

#### Freedom of hospitalization

Concerning freedom of hospitalization, S-ICD therapy showed no differences compared to TV-ICD therapy (HR 1.7; 95 % CI, 0.85–3.40, *p* = 0.134) with consistent results on weighted analysis (HR 1.20; 95 % CI, 0.75–1.90, *p* = 0.446) (Figure 2C and Supplementary Figure 1C). Here, a total of 33 hospitalizations occurred during the follow-up period of 1.5 years (20 TV-ICD and 13 S-ICD) in the unweighted group. The main reasons for hospitalization were cardiovascular in 22 cases (15 TV-ICD and 7 S-ICD) followed by device-related problems in 6 cases (1 TV-ICD and 5 S-ICD). Non-cardiac and other reasons for hospitalization were rare (4 for TV-ICD and 1 S-ICD).

#### Comorbidity predicted survival with Charlson Comorbidity Index

Patients with TV-ICD had relevant lower projected 1-year survival rates based on CCI system (TV-ICD 21 vs. S-ICD 53%, *p* = 0.058) (Table 1). In contrast, the KM estimated survival rate at one-year in the study cohort was 97.5 % and between patients with S-ICD and TV-ICD (94.3 vs. 98.8%, p for log rank = 0.152), which differed from the CCI projected survival rate indicating that the CCI may not be of profound predictive value in a defibrillator cohort.

#### Freedom of device-related events

During the follow-up period, a total of 17 appropriate ICD-shocks occurred in the unweighted study cohort. Fourteen TV-ICD patients and 3 S-ICD patients received appropriate shocks due to VT or VF without significant differences on Cox-Regression analysis between the two ICD types (unweighted HR 0.99, 95 % CI 0.26–3.8, *p* = 0.988 and weighted HR 1.2, 95 % CI 0.75–1.9, *p* = 0.446). Inappropriate shocks were rare (*n* = 3) and occurred in patients implanted with TV-ICD. Of these, two patients were inadequately shocked for rapidly conducted atrial fibrillation and one patient was inadequately treated for 1:1 conducted atrial flutter. Furthermore, device-related events combining appropriate and inappropriate therapy or system infection did statistically not differ between patients implanted with TV-ICD or S-ICD in the unweighted (HR 1.6, 95% CI, 0.46–5.7, *p* = 0.464) and weighted analysis (HR 0.71, 95 % CI 0.33–1.50, *p* = 0.391) (Figure 2D and Supplementary Figure 1D), respectively.

#### Device-associated complications

Device-associated complications were systemically assessed, while no statistical comparison was provided for these endpoints given the rare event rate. No device dysfunction was observed during the follow-up period. In S-ICD patients, device dysfunction due to programming (e.g., vector programming) was also not observed. A total of 2 lead complications occurred in TV-ICD patients (1 fracture, 1 insulation dysfunction) necessitating revision. No lead complications occurred in S-ICD patients. A total of 7 infections were observed: 2 lead infections (1 TV-ICD / 1 S-ICD) and 4 pocket infections (1 TV-ICD / 3 S-ICD). One patient with TV-ICD had both lead and pocket infection. These infectious complications led to 3 surgical revisions, one S-ICD patient with pocket infection was managed with antibiotic therapy.

## Discussion

### Main findings

This single-center analysis revealed no differences in the composite endpoint as well as survival, freedom of hospitalization or of device-associated outcomes alone in a real-world cohort comparing the subcutaneous ICD and the transvenous ICD. These results persisted even after careful adjustment using inverse probability weighting based on the propensity score. In addition, this is the first study to assess the Charlson Comorbidity Index in a real-life cohort comparing S-ICD and TV-ICD, revealing higher projected survival rates in S-ICD patients compared to TV-ICD patients, although interpretation of these differences may be regarded as hypothesis generating since the CCI has been developed primarily as a tool for adjusting the prognostic value of comorbidities in a statistical model.

### Survival

To date, there is no clinical study evaluating survival in S-ICD patients as a primary outcome (13, 32–35). In the randomized controlled PRAETORIAN trial, Knops and colleagues described death from any cause as secondary outcome demonstrating no statistically significant difference between patients with S-ICD and TV-ICD (HR = 1.23; 95 % CI. 0.89–1.70) (13). Comparable to our study results, the main cause of death was cardiac-non arrhythmic followed by non-cardiac causes. Of note, 22% of the TV-ICD patients died of SCD while in S-ICD patients SCD as a primary cause of death occurred in 26% (13). In our study, only one S-ICD patient died of SCD. Several clinical trials used propensity-score matching as primary statistical method to pseudo-randomize TV-ICD and S-ICD therapy in a real-life cohort (32–35). Their results, as well as the results from the PRAETORIAN trial, were incorporated in a recently published meta-analysis by Fong and colleagues revealing no significant difference in mortality between the two ICD types (36). Interestingly, the authors provided a pooled Kaplan-Meier analysis to investigate the survival probability. Visual inspection indicates divergence of the two curves after a 4-year follow-up favoring a better survival in patients with S-ICD, although differences were not statistically significant (36). The EFFORTLESS S-ICD registry was designed to obtain clinical outcome data in S-ICD patients implanted with early generation devices (10). Recently, long-term results have been published and demonstrate an encouraging 5-year survival rate of 90.7% (37). The UNTOUCHED trial was designed to evaluate the inappropriate shock rate in a more typical, contemporary S-ICD cohort (14% having chronic kidney disease) (14). Here, the one-year-survival rate was 94.9% (14), which is comparable to the one-year survival observed among S-ICD patients in our cohort. In contrast to the study population in the UNTOUCHED trial, our study cohort incorporated almost 50% patients with secondary preventive ICD indication. Most recently, the ATLAS trial (avoid transvenous leads in appropriate subjects) included 503 patients being randomized to receive either a TV-ICD or S-ICD to evaluate perioperative complications (38). The survival rate was 98.8% within the S-ICD cohort at 6 months in a relatively young study population with a mean age of 49 years (38).

### Charlson Comorbidity Index and polymorbidity

This is the first study to evaluate the Charlson Comorbidity Index (CCI) in a S-ICD study population. In accordance with several other defibrillator studies (39–43), we used the CCI to visualize and to correct for potential extracardiac comorbidities. We observed particularly high CCI scores (for S-ICD patients a median CCI of 4; for TV-ICD patients a median CCI of 5) in our study cohort compared to the existing literature (39, 40, 42). Bhavnani and colleagues, for instance, investigated early mortality (< 1 year after ICD implantation) in an elderly ICD population (age about 78 years) with a mean CCI of 2.8. Here, a CCI above 5 was associated with an incidence of 78% for early mortality (39). In a large cohort of CRT-recipients a CCI > 5 was also an independent predictor of mortality regardless of indication for ICD-therapy (40). However, Poupin et al. compared 121 elderly ICD-patients (mean age 78 years) in a 1:2 fashion with younger ICD-patients (mean age 66 years) as controls (42). In the elderly patients with a CCI of 4 or higher, the 5-year follow-up survival rate was 28 % and therefore significantly lower compared to elderly patients with lower CCI indices. In line with the younger ICD population included in our study, the mean survival rate of the control ICD population was remarkably higher with 72% (42) suggesting that increased age partially drives mortality in the context of interpreting high CCI indices. Although the reported studies questioned the appropriateness of ICD implantations in patients with CCI > 5 (39, 40) or even > 4 (42), the high survival rates observed in our study cohort (in contrast to the predicted survival rates by the CCI score) suggest that it would have been arguable to withhold ICD implantation from these young but polymorbid patients. Another reason for the encouraging clinical performance could have been S-ICD implantation per se, as this technique reduces electrode movement, lead-related complications and procedural complications like pneumothorax therefore reducing morbidity in total (38, 44). Consequently, our results might add value to the discussion about the guideline's class III indication for ICD-implantation in patients with a life expectancy of < 1 year (8, 9), which is very often difficult to assess and define in clinical practice.

### Hospitalization

In the PRAETORIAN trial, only hospitalization for heart failure was assessed as secondary endpoint (13). Concerning cardiac-non arrhythmic hospitalization no significant difference was observed between S-ICD and TV-ICD patients (13). In this study, we observed numerically higher freedom of hospitalization rates in TV-ICD patients compared to S-ICD patients, though this difference did not persist after adjustment on the propensity-score. A higher CCI score as well as an older age in the TV-ICD patient population might account for the observed differences in our study cohort. To date, there are no other clinical studies evaluating causes of hospitalization in patients with S-ICD and TV-ICD.

### Device-associated complications

Device-associated complications were distributed equally between the two ICD types in our study except for lead-related complications, although the relatively small number of patients in this cohort needs to be taken into account when interpreting the results. We did not observe technical or mechanical problems with S-ICDs leads, while one lead infection occurred in a S-ICD patient. This is in line with the results from Fong's meta-analysis observing significantly lower lead-related complications in S-ICD patients (RR = 0.14; 95% CI 0.07–0.29; *p* < 0.0001) (36) as well as according to data from the PRAETORIAN trail and Brouwers dual-center propensity score-matched cohort (32). Fong observed no significant difference in device-related complications (RR = 0.59; 95 % CI 0.33–1.04; *p* = 0.07) (36). In a single-center experience investigating 70 S-ICD patients vs. 197 TV-ICD patients on the endpoints of the PRAETORIAN study, no differences in device complications were observed (16). Of note, 30 % of the patients had a secondary preventive ICD-indication (16). This is also in line with our study results as 51% of our S-ICD patients received an ICD for secondary prevention (rate in TV-ICD patients: 45%). Su and colleagues evaluated safety of S-ICD vs. TV-ICD therapy concerning inappropriate shocks, device-related infections and survival in a recently published meta-analysis comprising 7 studies. Su described no differences in device-related infections between the two ICD groups (OR = 1.57; 95% CI: 0.67–3.68) (45). In contrast, data from the recently published Monaldi Registry comparing 607 patients either implanted with S-ICD or TV-ICD demonstrate significantly lower adjusted risk for ICD related infections (OR = 0.07; 95% C. I. 0.009-0.55; *p* = 0.01) (15). Preliminary analysis from the randomized-controlled ATLAS trial demonstrated superiority of the S-ICD regarding lead-related complications with a relative risk reduction of 92% (OR = 0.08; 95% C. I. 0.00–0.55; *p* = 0.003) (38, 46). In fact, we observed numerically more pocket infections in S-ICD patients in our study cohort. Accordingly, Rordorf et al. reported a significantly higher risk of pocket complications defined as hematoma, erosion or infection in S-ICD patients compared to patients with TV-ICDs (OR = 2.18; 95 %CI 1.30–3.66; *p* = 0.003) in a recently published meta-analysis by evaluation of 13 studies comparing S-ICD and TV-ICD therapy (47).

### Clinical implications

This real-world study investigated patients with primary and secondary preventive indication for ICD therapy receiving either a single chamber TV-ICD or a S-ICD and revealed that results for both ICD types with respect to the composite endpoint, survival, freedom of hospitalization, and freedom of device-associated complications did not differ. Additionally, we believe that non-lead related device complications can and will be further diminished in S-ICD patients as reported by the preliminary analysis of the ATLAS trial (38). Therefore, we provide additional evidence to recently published data from the PRAETORIAN trial in our all-comers study cohort of patients with primary and secondary preventive indication for ICD therapy and add clinical outcome data concerning survival and freedom of hospitalization to the existing literature. Further, this is the first study to evaluate S-ICD patients based on the CCI to correct for extracardiac comorbidities revealing a higher survival rate than predicted by the high CCI indices, at least in part, for relatively young ICD patients with increased burden of morbidity included in this study.

### Limitations

Our study is retrospective in nature, hence all potential limitations of such a design apply to this analysis. We aimed to minimize confounding by carefully adjusting data by performing a propensity-score based analysis. Despite this, residual confounding in observational studies cannot be entirely excluded, especially for unmeasured confounders. Balance of measured and unmeasured covariates is achieved only in randomized, placebo-controlled trials. Additionally, the low inclusion rate and low event number in our study as well as potential selection bias have to be taken into account when interpreting the results of this study. Although the results are in line with recent reports, there was a limited number of patients included in this study. Strengths of this study are detailed evaluation of clinical outcome data, incorporation of a real-world study cohort and evaluation of the Charlson Comorbidity Index, although the CCI has been evaluated but not validated in TV-ICD patients (39–43) or S-ICD patients.

## Conclusion

Our single-center observational study revealed no differences of the transvenous ICD compared to subcutaneous ICD regarding survival, freedom of hospitalization and device-associated complications in a real-world cohort. These results persisted even after careful adjustment for measured confounders using the Charlson Comorbidity Index and inverse probability weighting based on the propensity score. Of note, this is the first study to evaluate CCI in a S-ICD population demonstrating higher survival rates than predicted by the high CCI indices for young ICD patients with increased burden of morbidity included in this study.

## Data availability statement

The original contributions presented in the study are included in the article/Supplementary material, further inquiries can be directed to the corresponding author.

## Ethics statement

The studies involving human participants were reviewed and approved by IRB J. W. Goethe University/University Hospital. The patients/participants provided their written informed consent to participate in this study.

## Author contributions

BK, SH, MV, and JE contributed to conception and design of the study. FO, FH, FM, MV, and JE organized the database. BK, FB, and JE performed the statistical analysis and wrote the first draft of the manuscript. All authors contributed to manuscript revision, read, and approved the submitted version.

## Conflict of interest

Author JE reports receiving consultant fees, travel support and lecture fees from ZOLL Medical, travel grants from Bayer Vital, St. Jude Medical/Abbott, Novartis and lecture fees from Servier, Pfizer and Bayer Vital and was a fellow of the Boston Scientific heart rhythm fellowship program. Author MV reports consulting fees and/or non-financial support from Biotronik, Medtronic, and Pfizer, outside the submitted work. The remaining authors declare that the research was conducted in the absence of any commercial or financial relationships that could be construed as a potential conflict of interest.

## Publisher's note

All claims expressed in this article are solely those of the authors and do not necessarily represent those of their affiliated organizations, or those of the publisher, the editors and the reviewers. Any product that may be evaluated in this article, or claim that may be made by its manufacturer, is not guaranteed or endorsed by the publisher.
