# Supplementary material for: Clinical outcomes of subcutaneous vs. transvenous implantable defibrillator therapy in a polymorbid patient cohort
Source: Front Cardiovasc Med. 2022 Oct 18;9:1008311. doi: 10.3389/fcvm.2022.1008311 (PMC9624387; doi:10.3389/fcvm.2022.1008311)
Supplement: Supplementary file 1 [file Data_Sheet_1.DOCX]

## Supplementary Figure S1

**
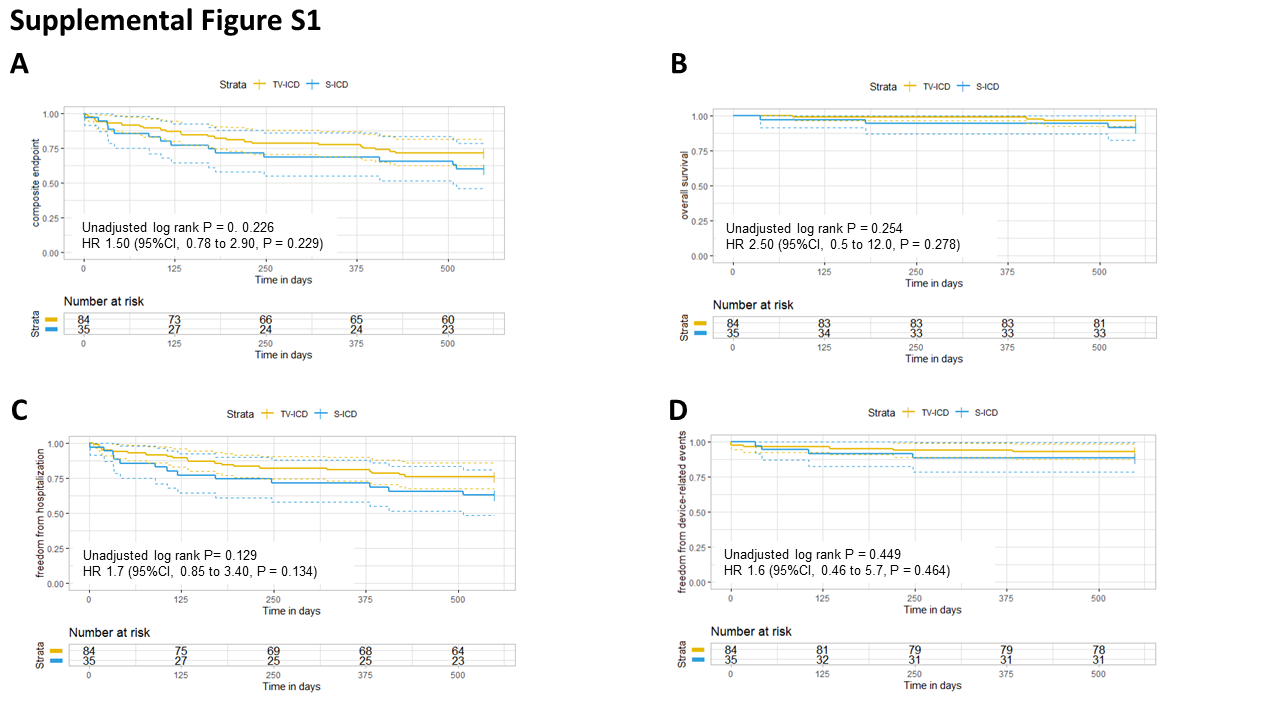
**

**Supplementary Figure S1.** Unadjusted Kaplan-Meier curves in patients with subcutaneous and transvenous ICD for (A) the composite endpoint, (B) overall survival, (C) freedom of hospitalization and (D) freedom of the device-related events at 1.5 year.
